# Depression and Quality of Life in Patients with Diabetes: A Systematic Review from the European Depression in Diabetes (EDID) Research Consortium

**DOI:** 10.2174/157339909788166828

**Published:** 2009-05

**Authors:** Miranda T Schram, Caroline A Baan, François Pouwer

**Affiliations:** 1 National Institute for Public Health and the Environment, Centre for Prevention and Health Services Research, Bilthoven, The Netherlands; 2 CoRPS--Center of Research on Psychology in Somatic diseases, Tilburg University, Tilburg, The Netherlands; 3 Department of Medical Psychology, EMGO Institute, VU University Medical Center, Amsterdam, The Netherlands

**Keywords:** Diabetes, Depression, Quality of life, Review.

## Abstract

Diabetes patients are known to have a worse quality of life than individuals without diabetes. They also have an increased risk for depressive symptoms, which may have an additional negative effect on their quality of life. This systematic review summarizes the current knowledge on the association between depressive symptoms and quality of life in individuals with diabetes. A systematic literature search using MEDLINE, Psychinfo, Social SciSearch, SciSearch and EMBASE was conducted from January 1990 until September 2007. We identified studies that compared quality of life between diabetic individuals with and without depressive symptoms. Twenty studies were identified, including eighteen cross-sectional and two longitudinal studies. Quality of life was measured as generic, diabetes specific and domain specific quality of life. All studies reported a negative association between depressive symptoms and at least one aspect of quality of life in people with diabetes. Diabetic individuals with depressive symptoms also had a severely lower diabetes specific quality of life. Generic and domain specific quality of life were found to be mild to moderately lower in the presence of depressive symptoms. Therefore, increased awareness and monitoring for depression is needed within different diabetes care settings.

## INTRODUCTION

Diabetes is a serious health problem in the Western world. According to the International Diabetes Federation, 189 million individuals have diabetes world wide [1]. The prevalence of diabetes in Western societies is rapidly rising; worldwide the number of individuals with diabetes is expected to have doubled in 2025. Diabetes is frequently accompanied by serious short term complications such as hypoglycaemia, but also by disabling long term complications like cardiovascular disease, neuropathy, nephropathy and retinopathy. Less known is the increased risk for depression: individuals with diabetes have a two-fold increased risk for depression, affecting approximately 1 in every five diabetes patients [2, 3]. Depressive symptoms are particularly common among diabetes patients with co-morbid health problems, as compared to patients with diabetes alone [4].

Diabetes care mainly consists of self care. Diabetes patients themselves have to regulate their blood glucose levels by monitoring their blood glucose levels and by balancing their food intake, physical activities and their intake of oral hypoglycaemic agents and/or insulin. The overall treatment goal is to prevent acute and chronic complications, while preserving a good quality of life. Several studies have shown that the quality of life in diabetes is decreased as compared to individuals without diabetes [5-7]. Furthermore, the presence of diabetic complications has an additional negative impact on quality of life [5, 8]. Depressive symptoms are known to have a considerable impact on quality of life as well [6]. The co-occurrence of depressive symptoms and diabetes may even further decrease quality of life. When this is indeed the case, this stressed the importance of an increased awareness and treatment of depressive symptoms within diabetes care. 

Depressive symptoms may thus be an important determinant of quality of life in diabetes. Therefore, a study on the impact of depressive symptoms on quality of life in individuals with diabetes is warranted. This systematic review aims to describe the current knowledge on the association of depressive symptoms with various aspects of quality of life in individuals with diabetes.

## METHODS

A literature search using the databases MEDLINE, Psychinfo, Social SciSearch, SciSearch and EMBASE was performed to identify published studies that evaluate the effect of depressive symptoms on quality of life in adult individuals with diabetes. Only full papers were included in this review, (meeting) abstracts and reviews were not included. The terms *diabetes* or diabetic* or *iddm (insulin dependent diabetes mellitus)* or *niddm (non-insulin dependent diabetes mellitus)* or *diabetes mellitus* were combined with *depression* or *depressive* or *depressed mood *or *depressed patients* or *depressed subjects* or *depressed women *or dysthym* or *dysthymic disorder* and with *quality of life *or* activities of daily living*. The search was limited to articles in English, published between January 1990 and September 2007. The resulting publications were screened for relevance, first by title and then by abstract (by MTS). To be included, studies had to compare quality of life in diabetic individuals with or without depression or depressive symptoms. Reference lists of the relevant studies were examined to obtain additional reports.

### Quality of Life

“Quality of life” represents an individual’s perception on the ability to function well on a physical, mental and social level. It can be measured in a reliable and valid manner by use of self-reported questionnaires, which can be categorised in three main groups; generic, disease specific and domain specific questionnaires. Generic questionnaires measure quality of life in general terms, independent of the presence of any disease. Disease specific questionnaires measure the consequences of a specific disease for the quality of life. Domain specific questionnaires focus on certain domains of quality of life, for instance physical inabilities. 

### Depression

Depression can be measured by use of a structured psychiatric diagnostic interview (gold standard) after which the diagnosis can be made according to criteria of the Diagnostic and Statistical Manual of Psychiatric Disorders (DSM). However, these interviews are time consuming, therefore depression questionnaires were developed. These questionnaires are often used as a screening instrument for depressive symptoms, as a first step in the diagnostic process or as outcome measures. Their validity is less than the diagnostic interview, but they do give an impression on the prevalence of depressive symptoms. 

### Diabetes

The gold standard to diagnose diabetes is an oral glucose tolerance test (OGTT). However, diabetes can also be diagnosed by measuring fasting glucose levels twice, as most physicians do today. Self-reported diabetes is a reliable measure of the presence of diabetes as well [9].

### Effect Size

We considered a <15%-point difference in quality of life between diabetic individuals with and without depressive symptoms as small, a 15 to 30%-point difference as moderate, and a difference >30%-points as a severe difference.

## RESULTS

The literature search yielded 496 studies. The titles of these 496 studies were screened for relevance, 354 studies were excluded as not relevant. Of the 142 remaining studies, 31 were excluded because these were reviews, 5 were excluded because these were animal studies, 29 were intervention studies comparing different treatments and 12 studies were only published as (meeting) abstracts. Of the 65 remaining studies the abstract and full text paper were screened. The large majority of studies (n=51) did not compare quality of life measures in diabetic individuals with or without depression or depressive symptoms and were therefore excluded. We found 14 studies that did compare quality of life between diabetic individuals with and without depression or depressive symptoms. These 14 studies were included in this review. From the reference lists of these studies we selected another 6 studies that met the inclusion criteria. In total 20 studies were included in this review.

Table **1** describes the study characteristics of the included studies. The majority of studies (18 out of 20) had a cross-sectional design. Only two studies investigated the effect of depressive symptoms in diabetic individuals on quality of life in a prospective setting [10, 11]. Half of the studies did not discriminate between type 1 and 2 diabetes. All studies, except Goldney *et al*. [16], had a study sample of ≥ 100 individuals with diabetes. 

### Definitions of Depression, Diabetes and Quality of Life 

There is a large variation in the methods used to assess depression and depressive symptoms. Three studies used a standardised diagnostic interview (the gold standard) to assess depression. All other studies used various questionnaires to assess depressive symptoms. 

Diabetes was mostly defined as diagnosed by a physician. Four studies used self-report to define diabetes. 

Quality of life was mainly determined by use of generic questionnaires. Generic quality of life was measured in thirteen studies. The Medical Outcomes Study 36-item Short Form Health Survey (SF-36) or an abbreviation of this questionnaire was used most frequently. The SF-36 contains eight multi item scales: physical function, role limitations due to physical health problems, bodily pain, general health perceptions, vitality, social functioning and role limitations due to emotional problems. The scores per item are linearly converted to a 0 to 100 scale, with higher scores indicating higher levels of functioning or well-being. Information on physical function, role physical, bodily pain and general health is combined into a physical components summary (PCS). Information on vitality, social functioning, role emotional and mental health is combined into a mental components summary (MCS). These summary scores are highly comparable between the SF-36 and the abbreviated versions of the SF. Diabetes specific quality of life was measured in only two studies [12, 13]. The questionnaires used focus on the impact and satisfaction of diabetes treatment and emotional responses to having diabetes. Six studies evaluated quality of life by use of domain specific questionnaires, mainly Activities of Daily Living (ADL) and Instrumental Activities of Daily Living (IADL) questionnaires. ADL represent activities as washing your self, dressing yourself, eating and drinking, using the toilet etc. IADL represents activities as shopping, cooking meals, walking two or three blocks, walking up and down ten steps, doing light housework etc.

### The Association of Depressive Symptoms with Generic Quality of Life

**Fig. (1):**
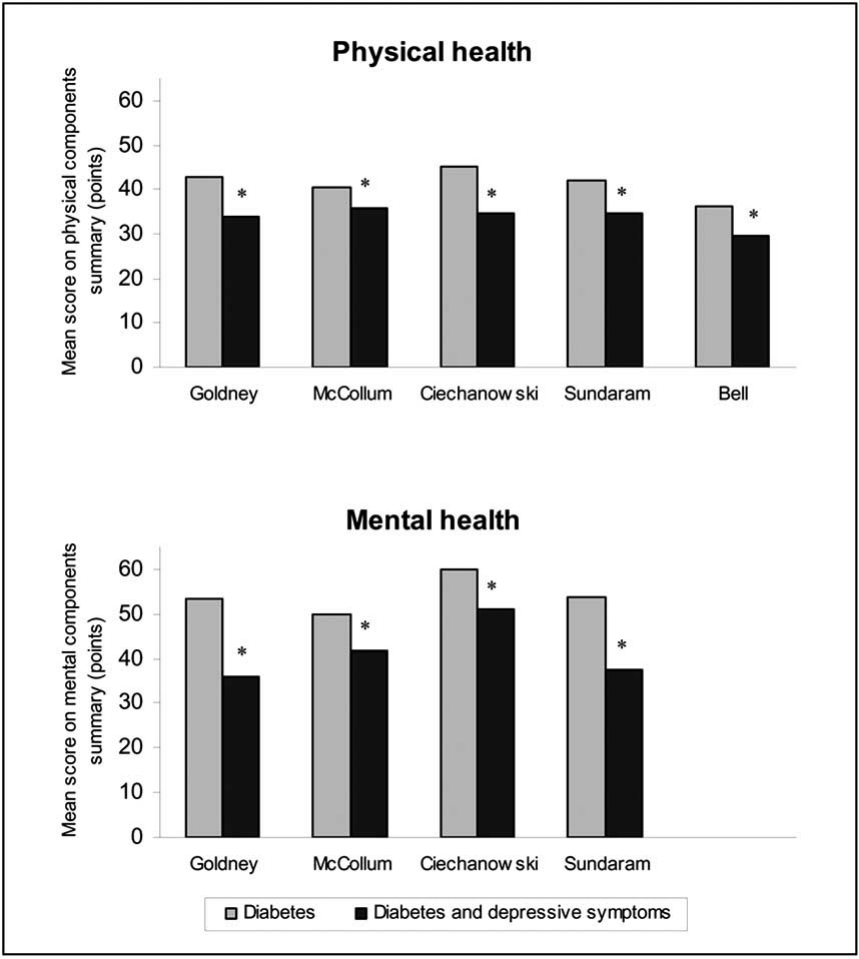
Difference in physical and mental health between diabetic individuals with and without depressive symptoms in studies using physical and mental components summary scores of the Short Form. * Significant difference between diabetic individuals with and without depressive symptoms.

In general, most studies showed a moderate, negative association of depressive symptoms on generic quality of life in individuals with diabetes [8, 12-22]. The majority of studies measured quality of both physical and mental health, albeit using different questionnaires. Five studies presented the absolute PCS scores [14, 16, 17, 20, 23], and four the MCS scores [14, 16, 20, 23] of the Short Form for diabetic individuals with and without depressive symptoms. These data are presented in Fig. (**1**) and show that diabetic individuals with depressive symptoms do consistently worse on both physical and mental health as compared to individuals with diabetes alone. These differences in both physical and mental health were mild to moderate. The results suggest a similar strength of the associations between depressive symptoms and both physical and mental health (Fig. **1**). 

The four studies that used more extensive versions of the Short Form Health Survey (SF-20 to SF-36) allowed a comparison based on the subscales of the Short Form; physical function, role function, overall health, social function, pain and mental health [12, 15, 16, 18]. This comparison shows that depressive symptoms were most strongly associated with role function and social function (moderate to severely poorer scores, Table **2**). Its associations with physical function, overall health and mental health were moderate, while pain was only mild to moderately lower among individuals with both depressive symptoms and diabetes. 

### The Association of Depressive Symptoms with Diabetes Specific Quality of Life

Four studies investigated the association of depressive symptoms with diabetes specific quality of life. These studies show a moderate to severely worse diabetes specific quality of life in the presence of depressive symptoms [12, 13, 24, 36]. Individuals with both diabetes and depressive symptoms were less satisfied with their treatment, experienced a greater impact of the treatment, worried more about the impact of diabetes in the future and about the social and vocational impact of diabetes. In addition to these studies, Kohen et al. [15] investigated the association of depressive symptoms with the number of hypoglycaemic events and other symptoms of diabetes, but did not find any difference between diabetic individuals with and without depressive symptoms.

### The Association of Depressive Symptoms with Domain Specific Quality of Life

Several studies have shown that both ADL and IADL are more impaired in diabetic individuals with depressive symptoms as compared to individuals with diabetes alone. Problems with ADL activities were reported more often by diabetic individuals with depressive symptoms as compared to individuals with diabetes alone. The difference in the prevalence of ADL problems between diabetic individuals with and without depressive symptoms was mild to moderate, ranging from 4.2 to 16.9% [11, 14, 21, 25]. IADL was severely worse in individuals with diabetes and depressive symptoms. The difference in prevalence of IADL problems between diabetic individuals with and without depressive symptoms was moderate, ranging from 13.3 to 27.6% [14, 25]. Functional limitations as measured by Egede *et al.* [25] appeared to be closely related to IADL. Individuals with both depression and diabetes reported 20% more functional limitations than individuals with diabetes alone. McCollum [14] also reported on cognitive problems and self-reported health and showed that the individuals with both depression and diabetes more frequently had cognitive problems (difference 20%) and their self-reported health was 13% lower as compared to individuals with diabetes alone. 

### Is Depression Causally Related to Functional Disability?

Few studies investigated the association of depression and depressive symptoms with quality of life in individuals with diabetes in a prospective setting. We found only two longitudinal studies, that focussed on domain specific quality of life. These two studies investigated whether depressive symptoms can predict the development of functional limitations in the future [10, 11]. Both studies show that depressive symptoms indeed predict the development of limitations in ADL and IADL activities. Individuals with depressive symptoms had a 41 to 89% increased risk to develop functional limitations. After multiple adjustments for behavioural and demographic confounders and co-morbidities, depression remained a significant predictor of problems with ADL activities in one study [11], while the association disappeared in the other [10]. 

### Magnitude of the Effect of Depression on Quality of Life in Diabetes

Wexler et al. compared the magnitude of the effect of depressive symptoms on quality of life in 909 individuals with diabetes directly with other factors that influence quality of life [8]. This study shows that depression was more strongly related to generic quality of life than microvascular complications, heart failure and the number of medications used.

## DISCUSSION

This review shows that depressive symptoms in individuals with diabetes are associated with a worse quality of life. The majority of studies show that generic, diabetes specific, as well as domain specific quality of life are poorer in the presence of depressive symptoms. Generic and domain specific quality of life were mild to moderately reduced in diabetic individuals with depressive symptoms, while diabetes specific quality of life was moderate to severely worse. In addition, there is evidence that depressive symptoms can predict the development of functional limitations in the future, suggesting a causal relationship between depressive symptoms and functional disability.

All studies included in this review show a negative association of depressive symptoms with at least one aspect of quality of life in individuals with diabetes. The consistency of this finding strongly supports the hypotheses that individuals that have both depressive symptoms and diabetes have a worse quality of life than individuals with diabetes alone. However, we also found differences in the effect size of depressive symptoms on quality of life. These differences may be explained by the use of different instruments to measure depressive symptoms, diabetes and quality of life and possible differences in the perception of quality of life between countries and ethnic groups.

Prospective studies on the association of depression and quality of life in individuals with diabetes are scarce. The available studies suggest that depression may indeed precede a decrease in quality of life [10, 11]. However, reversed causality, e.g. that a reduced quality of life or physical function in individuals with diabetes precedes the development of depressive symptoms, cannot be ruled out. Diabetes, depression and quality of life are closely interrelated. The causality and time path of these relations remains largely unknown. Evidence exists that diabetes is causally related to depression and vice versa. However, evidence on this area is weak. Therefore, longitudinal studies including estimates of diabetes, depression and quality of life are needed to elucidate their interrelations. 

Several factors are known to influence both depressive symptoms and quality of life, including age, sex, marital status, educational level or income. These factors may confound the association between depressive symptoms and quality of life. About half of the studies evaluated here did not take these factors into account by adjusting for them in statistical analyses. However, the studies that did take these factors into account, did not demonstrate large effects on the association between depressive symptoms with quality of life [8, 10-12, 14, 18, 21, 25]. This may suggest that the associations presented here will not be largely confounded by these factors. However, confounding is a major issue in epidemiology. The observation that about half of studies did not adjusted their analyses for confounding factors suggests that more elaborative studies on this subject are warranted.   

Both physical and mental quality of life scores were related to depressive symptoms. One may argue that psychiatric status is per definition related to mental health. Some studies indeed excluded the mental component of their quality of life questionnaire for this reason [12, 26].

Next to the considerable effect of depression on quality of life in individuals with diabetes, as demonstrated by this review, depression also contributes to poor self-care, poor adherence to medical treatment, higher rates of medical morbidity and mortality and increased health-care costs [23, 27-30]. Several studies have shown that glycemic control is worse in diabetic individuals with as compared to those without depressive symptoms [31]. In addition to this, the presence of depressive symptoms is also related to worse diabetes self-care, reflected by a worse adherence to diet and exercise advice, use of oral glucose lowering medication and frequency of glucose monitoring [23, 26, 37, 38]. Currently, the causality of these associations is not known since all studies are based on cross-sectional data. However, one study evaluated whether a stepped care approach for depressive symptoms in individuals with diabetes improved self-care. However, despite an improvement of depressive symptoms that exceeded the effect of usual care, no effect on self-care could be observed [37]. This study stresses the importance of an integrated diabetes care approach that addresses both the practical and emotional issues in diabetes.  

The presence of depression thus has major consequences for individuals with diabetes. However, several studies have shown that depression can be well treated in individuals with diabetes [32, 33]. Altogether, this review stresses the importance for intervention when individuals with diabetes present with depressive symptoms. However, only a small percentage of diabetic individuals is currently being recognised as being depressed in primary and secondary medical care settings [34]. Therefore, increased awareness for depression in diabetes care is needed. This can be achieved by including screening instruments for depression as part of regular diabetes care [13].

In conclusion, this review shows that higher levels of depressive symptoms are associated with an impaired quality of life in individuals with diabetes. Diabetes specific quality of life is severely lower among individuals with diabetes and depressive symptoms. Depressive symptoms may even predict the development of functional limitations. As a consequence, depressive symptoms jeopardize the ability of diabetic individuals to take care of themselves. Therefore, screening for and monitoring of depressive symptoms should be integrated in standard diabetes care. Furthermore, as most studies on this subject have a cross-sectional design; it is difficult to infer conclusions regarding the causality of the associations. More prospective studies are therefore needed to elucidate the causality of the association between depressive symptoms and quality of life in individuals with diabetes. 

## Figures and Tables

**Table 1:** Studies Evaluating the Association between Depressive Symptoms and Quality of Life in Individuals with Diabetes

Author (Publication Year)	Country	Study Design	N Diabetes	Measure for Diabetes	Type of Diabetes	Measure for Depression	Measure for Quality of Life		Effect of Depressive Symptoms	
Generic Quality of Life										
Jacobson (1997) [12]	US	Cross-sectional	240	Physician diagnosis	1&2	HSCL90 R	SF-36	Physical health	–	Moderate
Kohen (1998) [15]	UK	Cross-sectional	100	Physician diagnosis	1&2	HADS	SF-28		–	Moderate
Goldney (2004) [16]	Australia	Cross-sectional	97	Self-reported	1&2	PCEMD	SF-36	Physical health Mental health	– –	Small Moderate
Wexler (2006) [8]	US	Cross-sectional	900	Physician diagnosis	2	HADS	Health Utility Index		–	Large
McCollum (2007) [14]	US	Cross-sectional	1572	Physician diagnosis	1&2	ICD-9	SF-12	Physical health Mental health	= –	No effect Small
Hänninen (1999) [18]	Finland	Cross-sectional	222	Physician diagnosis or fasting glucose levels	2	Zung DS	SF-20		–	Moderate
Kaholokula (2003) [35]	Hawaii	Cross-sectional	146	WHO criteria 1999, c-peptide	2	CES-D	SF-36	Physical health	–	Moderate
Ciechanowski (2000) [23]	US	Cross-sectional	367	Physician diagnosis	1&2	HSCL90 R	SF-12	Physical health Mental health	– –	Small Small
Sundaram (2007) [20]	US	Cross-sectional	385	Physician diagnosis	2	CES-D	SF-12	Physical health Mental health	– –	Small Moderate
Eren (2008) [22]	Turkey	Cross-sectional	108	Physician diagnosis	2	DSM IV, HRDS	WHO QOL-BREF		–	Moderate
Paschalides (2004) [19]	UK	Cross-sectional	184	Physician diagnosis	2	Well-Being Questionnaire	SF-36	Physical health Mental health	– –	Moderate Large
Pawaskar (2007) [21]	US	Cross-sectional	792	Physician diagnosis	2	CES-D	SF-12		–	Moderate
Bell (2005) [17]	US	Cross-sectional	696	Physician diagnosis	1&2	CES-D	SF-12		–	Small
**Disease Specific Quality of Life**										
Jacobson (1997) [12]	US	Cross-sectional	240	Physician diagnosis	1&2	HSCL90 R	DQOL		–	Large
Hermanns (2006) [13]	Germany	Cross-sectional	376	Physician diagnosis	2	CIDI	PAID	–	–	Moderate
Pouwer (2005) [36]	The Netherlands, Croatia and UK	Cross-sectional	539	Physician diagnosis	1&2	CES-D	PAID		–	Moderate
**Domain Specific Quality of Life**										
Black (1999) [24]	US	Cross-sectional	363	Physician diagnosis	2	CES-D	ADL IADL		– –	Moderate Moderate
Gregg (2002) [10]	US	Longitudinal	527	Self-reported	1&2	GDS	Functional limitations	–		Small
Egede (2004) [25]	US	Cross-sectional	1794	Self-reported	1&2	CIDI-SF	Functional limitations		–	Moderate
Bruce (2005) [11]	Australia	Longitudinal	1294	Physician diagnosis	2	GHS	ADL Mobility		– =	Small No effect
McCollum (2007) [14]	US	Cross-sectional	1572	Physician diagnosis	1&2	ICD-9	ADL IADL Cognitive limitations Physical limitations Self-reported health		= = – – –	No effect Small Moderate Moderate Small
Pawaskar* (2007) [21]	US	Cross-sectional	792	Physician diagnosis	2	CES-D	ADL IADL		– –	Unclassified Unclassified

– negative association of depression with quality of life

= no effect

Depression questionnaires: HSCL90R, Hopkins Symptoms Checklist 90-Revised, HADS, Hospital Anxiety and Depression Scale, PCEMD, Primary Care Evaluation of Mental Disorders questionnaire, ICD-9, International Classification of Diseases 9th revision, Zung DS, Zung Self-Rated Depression Scale, CESD, Center for Epidemiologic Studies Depression Scale, DSM IV, Diagnostic and Statistical Manual of Mental Disorders VI, HRSD, Hamilton Rating Scale for Depression, CIS-R, Clinical Interview Schedule, GDS, Geriatric Depression Scale, CIDI-SF, Composite International Diagnostic Interview Short Form, GHS, General Health Status questionnaire.

Quality of life questionnaires: SF-36, Medical Outcomes Study 36-item Short Form Health Survey, WHO QOL-BREF, World Health Organisation Quality of Life Assessment Brief version, DQOL, Diabetes Quality of Life Measure, PAID, Problem Areas in Diabetes, ADL, activities of daily life, IADL, instrumental activities of daily life.

* This study had a longitudinal study design, but the association of depression with quality of life was investigated cross-sectionally. The longitudinal analyses evaluated the predictors of depression. Results in this study were reported as number of ADL or IADL limitations, and could therefore the effect size could not be classified.

**Table 2:** Difference in Specific SF Scores between Diabetic Individuals with and without Depressive Symptoms from Four Studies that Used SF-20 to SF-36

Author	Percentage Lower SF-Score in Individuals with Diabetes and Depressive Symptoms as Compared to Individuals with Diabetes Alone				Range of Lower SF-Score	Effect Size
	Jacobson (1997) [12]	Kohen (1998) [15]	Hänninen (1999) [18]	Goldney (2004) [16]		
**Physical function **	-17 *	-30 *	-17 *	-26 *	-17 to -30%	Moderate
**Role function **	-28 *	-26 *	-100 *	-42 *	-26 to -100%	Moderate to severe
**Overall health **	-27 *	-16 *	-17 *	-26 *	-17 to -27%	Moderate
**Social function **	-19 *	-19 *	-40 *	-36 *	-19 to -40%	Moderate to severe
**Pain**	-15 *	-4.4	-25 *	-24 *	-4 to -25%	Mild to moderate
**Mental health**	-	-17 *	-20 *	-30 *	-17 to -30%	Moderate

* SF-Scores were significantly lower among individuals with diabetes and depressive symptoms as compared to individuals with diabetes alone. Jacobson *et al.* and Goldney *et al.* used the SF-36, Kohen *et al.* the SF-28 and Hänninen *et al.* the SF-20.
